# Computational analysis of potential candidate genes involved in the cold stress response of ten *Rosaceae* members

**DOI:** 10.1186/s12864-022-08751-x

**Published:** 2022-07-16

**Authors:** K. Mohamed Shafi, Ramanathan Sowdhamini

**Affiliations:** 1 National Centre for Biological Sciences (TIFR), GKVK Campus, Bangalore, Karnataka 560065 India; 2 The University of Trans-Disciplinary Health Sciences & Technology (TDU), Yelahanka, Bangalore, Karnataka 560064 India; 3 Molecular BIophysics Unit, Indian Institute of Science, 560012 Bangalore, India

**Keywords:** Rosaceae, Cold stress, Syntelog, Gene duplication, Gene promoter, Transcription factor, AP2/ERF family

## Abstract

**Background:**

Plant species from Rosaceae family are economically important. One of the major environmental factors impacting those species is cold stress. Although several Rosaceae plant genomes have recently been sequenced, there have been very few research conducted on cold upregulated genes and their promoter binding sites. In this study, we used computational approaches to identify and analyse potential cold stress response genes across ten Rosaceae family members.

**Results:**

Cold stress upregulated gene data from apple and strawberry were used to identify syntelogs in other Rosaceae species. Gene duplication analysis was carried out to better understand the distribution of these syntelog genes in different Rosaceae members. A total of 11,145 popular abiotic stress transcription factor-binding sites were identified in the upstream region of these potential cold-responsive genes, which were subsequently categorised into distinct transcription factor (TF) classes. MYB classes of transcription factor binding site (TFBS) were abundant, followed by bHLH, WRKY, and AP2/ERF. TFBS patterns in the promoter regions were compared among these species and gene families, found to be quite different even amongst functionally related syntelogs. A case study on important cold stress responsive transcription factor family, AP2/ERF showed less conservation in TFBS patterns in the promoter regions. This indicates that syntelogs from the same group may be comparable at the gene level but not at the level of *cis*-regulatory elements. Therefore, for such genes from the same family, different repertoire of TFs could be recruited for regulation and expression. Duplication events must have played a significant role in the similarity of TFBS patterns amongst few syntelogs of closely related species.

**Conclusions:**

Our study overall suggests that, despite being from the same gene family, different combinations of TFs may play a role in their regulation and expression. The findings of this study will provide information about potential genes involved in the cold stress response, which will aid future functional research of these gene families involved in many important biological processes.

**Supplementary Information:**

The online version contains supplementary material available at 10.1186/s12864-022-08751-x.

## Background

Rosaceae family is the third most economically important plant family after Poaceae (grasses) and Fabaceae (legumes) [1]. It includes some of the most widely produced edible fruit species like pome fruits from Maloideae [2] (e.g. apple and pear), stone fruits from Prunoideae [3] (e.g. peach, cherry, plum, almond) and berries from Rosoideae [4] (e.g. strawberry and raspberry) subfamilies, as well as important ornamental and timber species. Abiotic stresses affect plant development, growth and decrease their productivity. Plants respond to these environmental conditions by developing various physical, biochemical and genetic strategies. Substantial efforts have been made over the last few decades to decode plant molecular mechanisms in reaction and adaptation to various stresses. At the agricultural, genetic and molecular research levels, important traits such as fruit size, shape and flavour, yield and plant response to either biotic or abiotic stress are being targeted in order to improve traditional breeding [5]. Advances over the past few years in genomics and bioinformatics of Rosaceae have provided new opportunities to identify information in the level of genes responsible for their development [6].

Many abiotic stresses like cold, drought, salinity and heat have an impact on plant growth, development and agricultural productivity. Temperature is one of the most important environmental factor, which could regulate growth and development of the plant [7]. Plants have a repertoire of machinery to combat these stresses and counteract them by repressing or inducing expression of a series of response factors with diverse functions. An important group of these regulatory proteins is transcription factors (TFs), which help the plant to survive abiotic stress by affecting regulatory networks and plant development signalling pathways [8]. Plants from Rosaceae family are often grow in cold condition and are subjected to low temperatures [9]. It is important to understand the mechanism and distribution of genes involved in the cold stress response in these species. Plants reprogram their genes through regulatory mechanisms (transcriptional, post-transcriptional, and post-translational modifications) in response to cold stress. Therefore, studying the regulatory mechanisms involved in response and adaption to cold stress is pivotal to improve cold tolerance in plants [10].

In response to cold stress, several proteins such as dehydrins, heat-shock proteins and cold-regulated proteins are also involved in membrane stabilisation [11]. The finding of Arabidopsis C-repeat-binding factors (CBFs) which is an AP2/ERF transcription factor, helped in better understanding the gene regulatory mechanisms in response to cold [12, 13]. DRE/CRT/LTRE (dehydration responsive element/C-repeat/low temperature responsive element) *cis*-elements are mostly found in the promoters of many cold stress response genes and has been proven necessary for gene transcription under cold stress [14, 15]. This sequence is the recognition site for the CBF/DREB family of transcription factors, which bind and activate cold-responsive genes [16, 17]. The CBF transcription factor genes are also a part of the cold regulon and are induced in response to cold, and their induction is regulated by components upstream in cold response pathways [18, 19]. In addition, there are many other TFs and regulators, such as MYB, WRKY, NAC, SIZ1 and HOS1, which have key roles in cold stress tolerance [10]. These genes are direct or indirect players in the crucial role of protecting plants against cold stresses [20].

With next-generation sequencing (NGS) techniques, knowledge in the field of plant science has advanced. The ability to sequence transcriptome using RNA-seq has enabled a large-scale comparative analysis of many plants under different conditions such as abiotic stresses. There are few such reports available for Rosaceae plants in response to cold. A transcriptome study on strawberry identified candidate genes and revealed diverse regulatory network that responded to cold stress [21]. Another study on apple identified differentially expressed genes (DEGs) during cold stress at various intervals [22]. In addition to these, few other findings on genes involved in chilling and freezing stress and study on their regulatory network for peach and almond [10, 23] were also reported.

There are several gene families, which share highly conserved genome sequences with each other among the related species, as well as other taxonomic families. Even though many Rosaceae genomes are sequenced recently, a detailed study on cold regulated genes across these species has not been reported. In this study, we aim for a genome wide analysis of cold regulated genes and their promoter region in Rosaceae family species by focusing on ten plants within this family. Cold upregulated genes information for apple and strawberry obtained from the literature was used to investigate putative genes in other Rosaceae species. In addition, *cis*-elements in the promoter region of gene was compared. The findings from our study will pave the way for the comprehensive analysis and the understanding the mechanism of cold stress tolerance of these plants. This type of research can be expanded to other plant families and for different stress responses, resulting in a list of genes that can be targeted further.

## Results

### Cold stress upregulated genes in Rosaceae species

**Fig. 1:**
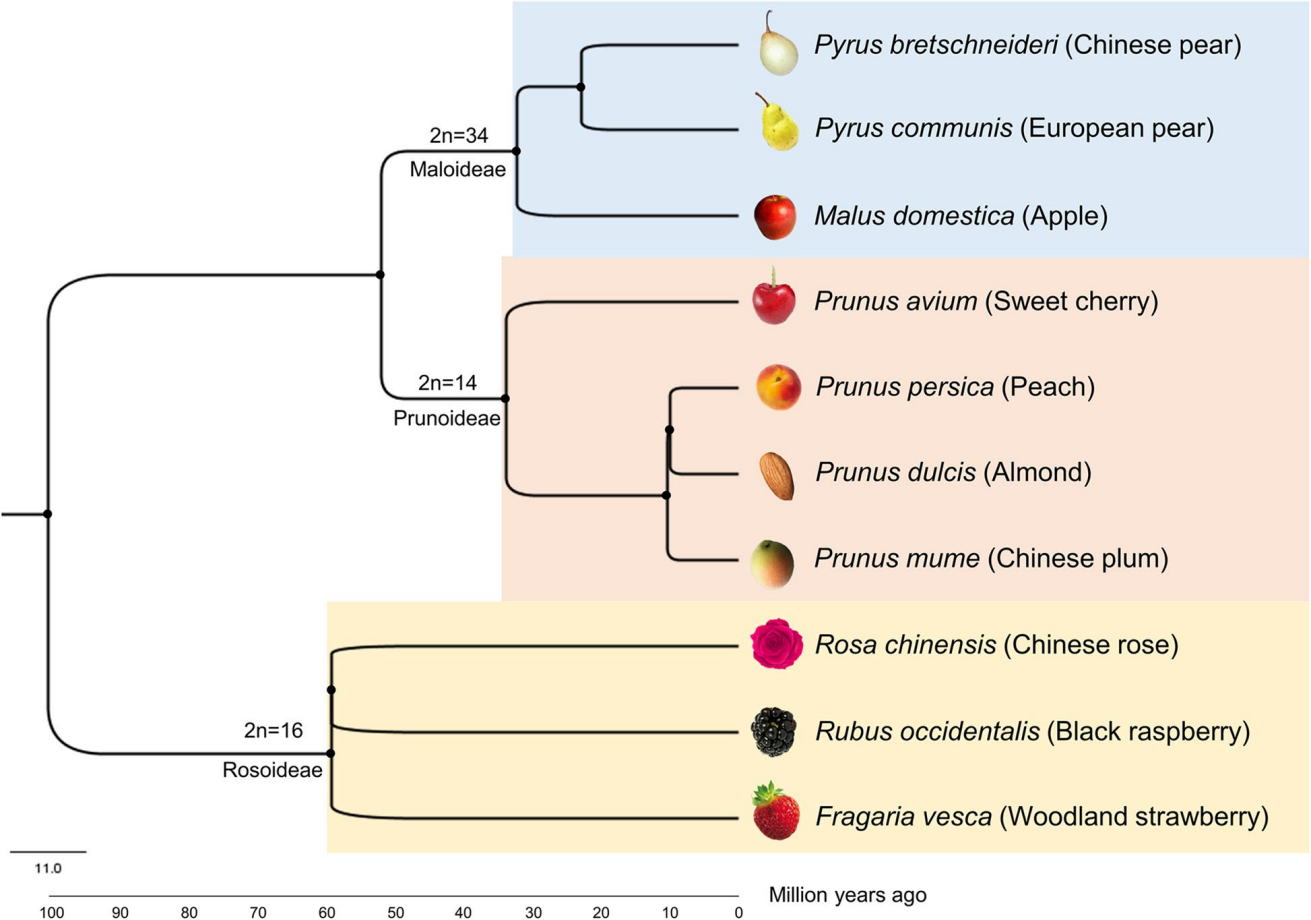
Plant species from Rosaceae family selected for this study. Phylogenetic tree depicts the estimated divergence time (in million years) for each species using TimeTree

In this study, ten plant species from Rosaceae family was selected based on their availability of genome sequence and chromosome information. For cold stress upregulated gene information, species belongs to the subfamilies Maloideae (*M. domestica*, *P. communis* and *P. bretschneideri*), Rosoideae (*F. vesca*, *R. chinensis* and *R. occidentalis*) and Prunoideae (*P. persica*, *P. avium*, *P. dulcis* and *P. mume*) were surveyed. A study from Zhang et al. [21] on transcriptome analysis to identify cold stress response genes in strawberry reported 901 upregulated DEGs. Another transcriptome study from Fan Du et al. [22] on apple identified 1883 cold stress upregulated genes. For both plants, a total 2784 differentially upregulated genes information was obtained from literature. Separately, we obtained genome sequence and chromosome information for each species from various databases (Fig. 1, Additional file 1).

Functions of cold-stress upregulated genes from both *M. domestica* and *F. vesca* were investigated and GO terms obtained from the homologous sequences. An enrichment analysis using these GO terms identified terms such as ‘response to salt stress’, ‘response to water deprivation’, ‘response to abscisic acid’ and ‘response to cold’. Various DNA-binding and kinase domains were also significantly enriched in functional domain and enrichment analysis (Additional file 2). These genes were then used to identify potential cold-stress responsive genes in eight other species from Rosaceae family.

### Identification of syntelogs and gene duplication analysis

**Fig. 2:**
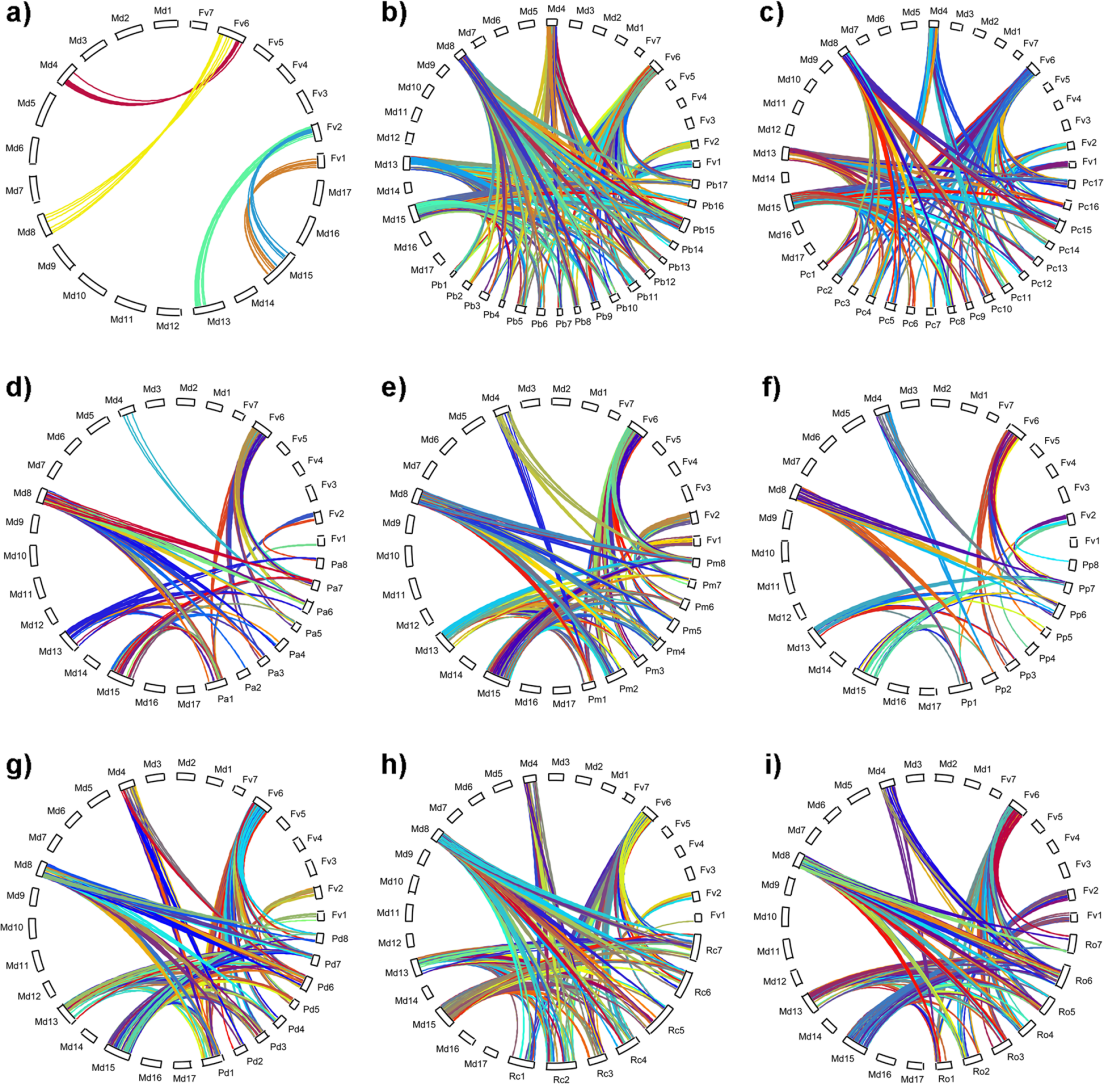
Inter species collinear blocks between each chromosome of **a**) *F. vesca* and *M. domestica* with **b**) *P. bretschneideri,* **c**) *P. communis,* **d**) *P. avium,* **e**) *P. mume,* **f**) *P. persica,* **g**) *P. dulcis,* **h**) *R. chinensis* and **i**) *R. occidentalis*. Each line indicates syntenic relationship and different colours, generated randomly, represents different synteny blocks

Syntelogs (fusion of homologue and synteny) were predicted across Rosaceae species using cold stress DEGs from *F. vesca* and *M. domestica*. Syntenic and collinear gene pairs between each species were identified using MCScanX program. It uses homologous gene pairs and gene co-ordinates in the chromosome to identify collinear blocks across species. A total of 313,768 protein sequences were obtained from genome data for Rosaceae species and all-versus-all BLAST searches were performed. Co-ordinates of each sequence were collected from annotation and provided to MCScanX algorithm along with homologue gene pairs from BLAST. The program detected syntelogs for all species and we selected 32 syntelog groups based on the presence of DEGs from *F. vesca* and *M. domestica* in each group. These groups include 1469 genes from different Rosaceae species (Fig. 2). An analysis of these groups showed that 35 genes from *F. vesca* (of chromosomes 1, 2 and 6) retain a collinear relationship with 37 genes from *M. domestica* (of chromosomes 4, 8, 13 and 15). A higher number of syntelog genes were observed for *Maloideae* species (*P. bretschneideri*-305 and *P. communis*-231) compared to other subfamily species. However, two Prunoideae species (*P. persica-45* and *P. avium-61)* identified comparatively low number of syntelogs. In order to understand the distribution of these genes among Rosaceae, physical location in the chromosomes were compared. The chromosome-wise distribution indicates that these genes are mostly distributed evenly among chromosomes of respective species (Additional file 3). The syntelog distribution among various subfamilies led us to examine the degree of gene duplication in the dataset.

Genes arising out of different duplication events like WGD, tandem, proximal or dispersed and singletons were classified into different categories using MCScanX program (Table 1). We observed more than 50% of the syntelogs in *P. bretschneideri*, *P. mume, R. chinensis* and *R. occidentalis* have been duplicated and retained from WGD events. Whereas in *P. dulcis, P. communis and P. avium,* the retained genes were 32.6, 27.7 and 19.7%, respectively. No WGD events was identified from *F. vesca*, *M. domestica* and *P. persica*. However, the proportions of dispersed duplication in *F. vesca*, *M. domestica*, *P. dulcis*, *P. communis, P. avium* and *P. persica* were considerably higher than other species. From the selected set of 37 genes from *M. domestica*, 54% were dispersed duplication. Around 45% of the genes from *F. vesca* was singletons.

**Table 1:** Number and percentage of duplications calculated for 32 syntelog group genes from different plants as classified by duplicate gene classifier

Species	Number of genes	Number of duplications (percentage)				
		WGD/Segmental	Dispersed	Proximal	Tandem	Singleton
*F. vesca*	35	0 (0)	10 (28.5)	5 (14.3)	4 (11.4)	16 (45.7)
*M. domestica*	37	0 (0)	20 (54)	5 (13.5)	2 (5.4)	10 (27)
*P. avium*	61	12 (19.7)	16 (26.2)	18 (29.5)	15 (24.6)	0 (0)
*P. bretschneideri*	305	157 (51.5)	18 (5.9)	69 (22.6)	60 (19.7)	1 (0.3)
*P. communis*	231	64 (27.7)	43 (18.6)	80 (34.6)	43 (18.6)	1 (0.4)
*P. dulcis*	196	64 (32.6)	18 (9.2)	40 (20.4)	60 (30.6)	4 (2)
*P. mume*	208	107 (51.4)	11 (5.3)	36 (17.3)	51 (24.5)	3 (1.4)
*P. persica*	45	0 (0)	11 (24.4)	13 (28.9)	16 (35.6)	6 (13.3)
*R. chinensis*	202	108 (53.5)	12 (5.9)	36 (17.8)	41 (20.3)	5 (2.5)
*R. occidentalis*	210	112 (53.3)	10 (4.8)	47 (22.4)	39 (18.6)	2 (0.9)

### Functional annotation and enrichment analysis

Syntelog gene functions were investigated by performing BLASTP and HMMSCAN against annotated plant sequences from *Viridiplanta*e clade. Each of the 32 groups is associated with a distinct gene family and comprises of at least one or more genes from each of the Rosaceae species (Additional file 4). We identified two groups of AP2/ERF transcription factor classes consisting of 115 genes in total from different Rosaceae species. These genes have a central AP2 functional domain. CBF proteins from AP2 family act as a key regulator in the cold signalling pathway. Another group of transcription factor WRKY, known to regulate either positively or negatively to cold stress, was observed. SQUAMOSA-promoter binding protein (transcription factor involved in the control of early flower development) was also present. Dehydrin COR genes, a multi-family of cold-regulated proteins present in plants, produced in response to cold and drought stress, was found in these groups. Two groups of cytochrome family of genes were present. These genes may involve directly or indirectly in the response to cold stress. Apart from these groups, few kinases, proteases and phosphatases were also part of syntelog groups. HMMSCAN was performed against Pfam database to verified the functions and domain architecture for each gene.

**Fig. 3:**
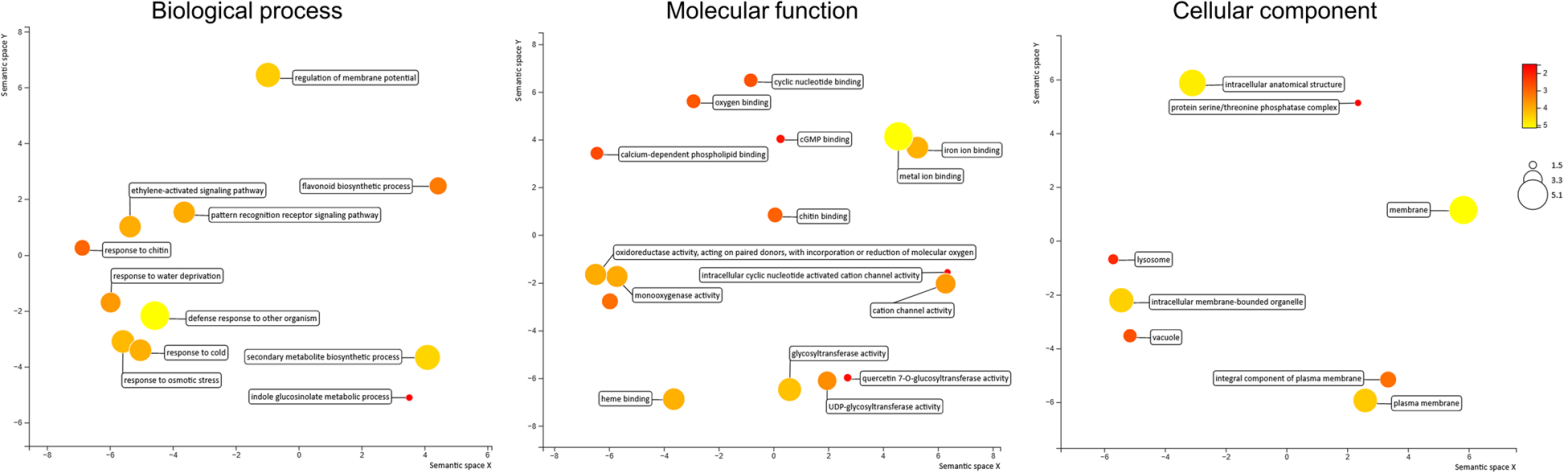
Scatter plots for significant GO terms from the categories of biological process, molecular function and cellular component enriched in the syntelog genes from 32 groups: The scatterplot shows the cluster of enriched terms in two-dimensional space (semantic *x* and *y* axes corresponding the log size value). Bubble colour and size indicates the log10 *p*-value of the GO term

A functional enrichment analysis was carried out using the GO terms derived from the homologous sequences and depicted in a scatter plot (Fig. 3). In biological process, majority the genes were involved in oxidation-reduction process. Notably, a higher enrichment for GO term ‘cold response’ with significant log size *P*-value was observed. Also, abiotic stress related terms like ‘response to water deprivation’ and ‘response to osmotic stress’ were significantly enriched in the syntelog genes. Ethylene activated signalling pathway related genes were also abundant in the groups. The role of ERF genes under cold stress has been reported in earlier studies. It can regulate gene expression either negatively or positively. Various molecular functions such as oxidoreductase activity, cation channel activity and ion binding activities were also enriched in this group of genes.

### Identification and classification of Transcription Factor Binding Sites (TFBS)

In plants, various TFs such as MYB, AP2/EREBP, bZIP, bHLH/MYC, HSF, NAC, HB and WRKY have been shown to regulate abiotic stress response. We obtained 1000 base pair upstream region for each syntelog gene using the coordinates from the genome annotation data. STIF algorithm (STIFAL) identified 11,145 TFBSs from the promoter sequence of 1408 syntelog genes after filtering false positives hits. We analysed the distribution of TFBS predicted in the promoter of each syntelog gene and compared the frequency predicted for each TF classes across species. A greater number of certain TFBSs than others was observed, could be partly due to the differences in the length of these *cis*-elements. We classified these TFBSs into different transcription factor families such as MYB (6126 number of occurrences), AP2/EREBP (776), bHLH (991), bZIP (735), ARF (728), WRKY (901), NAC (634), HSF (123), HB (60), and ABI3/VP1 (2). In general, MYB showed higher occurrences in the promoters, following to bHLH, WRKY, AP2, bZIP, ARF and NAC families (Table 2, Additional file 5). For *F. vesca* and *M. domestica*, 35 genes each were analysed and predicted 238 and 185 TFBS, respectively. We observed a slight increase in ARF, bHLH and WRKY binding sites in *F. vesca* compared to *M. domestica.* Whereas, *M. domestica* showed an increase in AP2 and MYB binding sites.

**Table 2:** Transcription factor binding sites predicted in the 1000 bp upstream region of the genes using STIFAL with a cut-off > 1.5 and their abundance in 10 different species. The number of occurrences of each TFBS at both family and subfamily level has been indicated in the table

**TFBS Statistics**		***F. vesca***	***M. domestica***	***P. avium***	***P. bretschneideri***	***P. communis***	***P. dulcis***	***P. mume***	***P. persica***	***R. chinensis***	***R. occidentalis***
Number of genes with TFBS		34	35	57	297	228	177	200	42	189	201
Number of genes without TFBS		1	0	4	8	3	6	7	3	10	8
Predicted TFBS		238	285	426	2265	1654	1464	1584	305	1430	1494
**TF family**	**TF subfamily**	**TFBS predicted in 1000 bp upstream ≥ 1.5 Z-score**									
MYB	Myb_box1	16	31	53	245	184	164	162	36	154	164
	Myb_box2	16	26	29	186	145	88	142	26	119	118
	Myb_box3	16	21	26	163	92	103	93	17	103	113
	Myb_box4	8	11	24	88	55	64	69	13	53	45
	Myb_box5	68	81	97	564	424	382	393	76	345	415
bHLH	G_box	12	6	19	103	78	75	71	8	73	55
	N_box	10	11	24	104	83	64	67	17	65	46
AP2/EREBP	DREB	9	13	24	108	90	67	78	14	69	65
	GCC_box	6	14	7	49	53	31	23	3	30	23
WRKY	W_box	24	21	33	184	142	111	124	28	103	131
bZIP	G_box1	1	2	1	5	3	4	4	0	6	3
	G_box2	13	15	20	105	81	61	87	13	85	68
	C_ABRE	2	5	3	35	27	19	22	3	19	23
	G_ABRE	0	3	3	18	12	7	8	0	10	8
ARF	AuxRE	22	10	29	136	86	108	129	22	91	95
NAC	Nac_box	12	13	23	123	80	82	93	23	87	98
HSF	HSE1	3	1	9	33	10	23	13	5	10	16
HB	HBE	0	1	2	15	8	11	6	1	8	8
ABI3/VP1	ABRE	0	0	0	1	1	0	0	0	0	0

AP2/ERF transcription family is the key regulator in cold signalling pathway. During cold stress, CBF/DREB TFs will bind to the *cis*-elements in the promoter of CORs and activate the pathway. In our study, we predicted a total of 776 AP2 binding sites (GCC-box and CRT/DRE) across all species. Around 50% of the syntelog genes of *M. domestica* has AP2 binding site in the promoter, which is highest, compared to other species (around 30–40%). *F. vesca* syntelog genes showed less AP2 binding site abundance (25%) in the promoters. In the promoters of a few genes, we observed a cascade of AP2 binding sites. UDP-Glycosyltransferase gene from *P. bretschneideri* predicted a cascade of seven AP2 binding sites (bHLH~AP2 ~ AP2 ~ AP2 ~ AP2 ~ AP2 ~ AP2 ~ AP2) in the promoter. B-Box domain protein from *P. communis* (pycom08g04150) predicted nine repeated AP2 TFBS along with other binding sites (bHLH~MYB ~ MYB ~ MYB ~ MYB ~ MYB ~ MYB ~ bZIP~AP2 ~ AP2 ~ AP2 ~ AP2 ~ AP2 ~ AP2 ~ AP2 ~ AP2 ~ AP2). These repeated AP2 binding sites were predicted within 200 bp upstream, six of them were GCC-box. There were many instances having more than five AP2 *cis*-elements repeats in the promoters of the genes. Interestingly, the AP2/ERF genes from *P. mume* (Pm020604) and *P. dulcis* (Prudul26A014155P1) showed repeated AP2 binding sites in their promoters. These genes could be playing an important role in the regulation of cold stress genes.

### Clustering and comparison of transcription factor binding sites among syntelogs

**Fig. 4:**
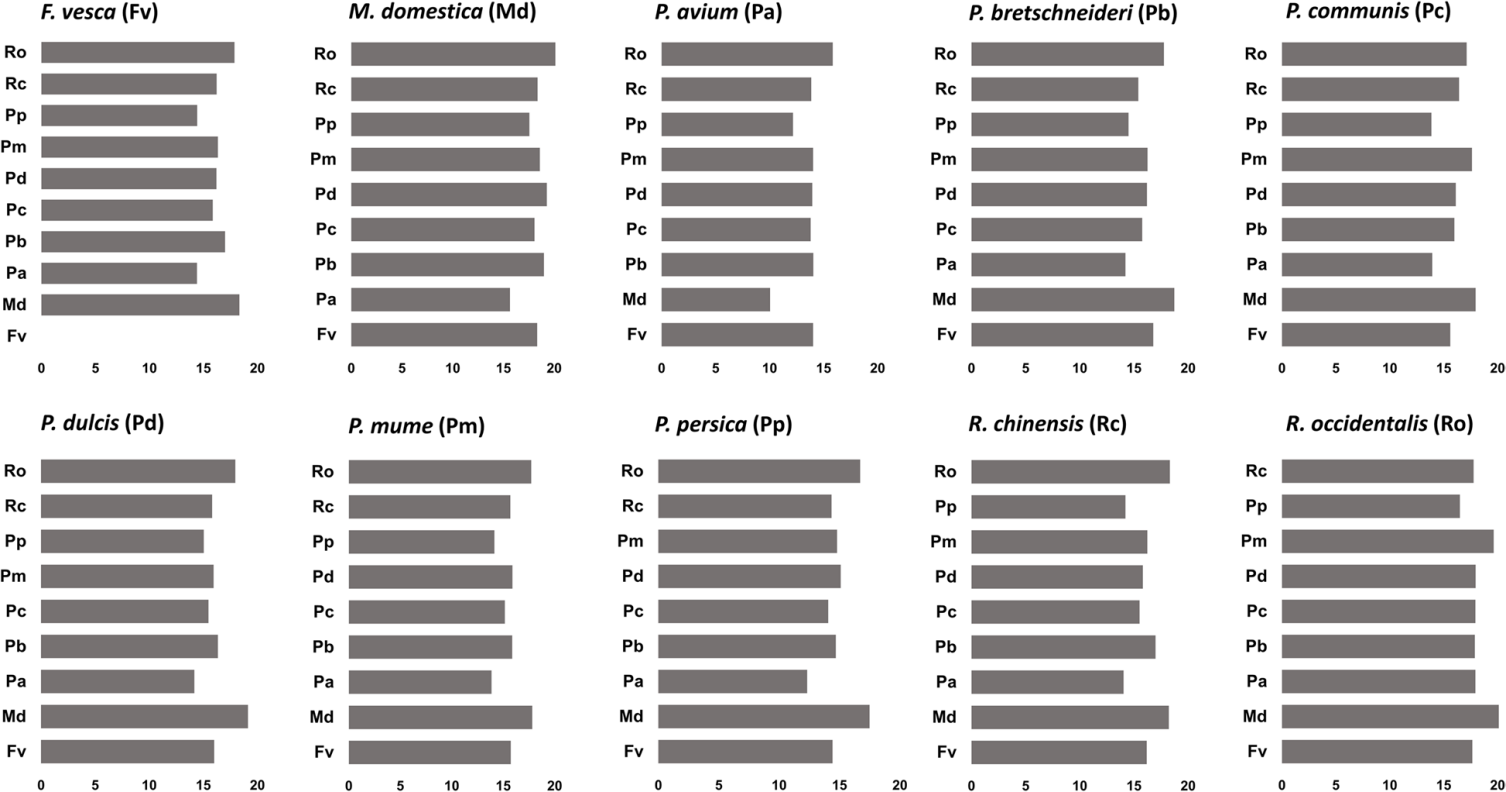
Percentage of ADASS scores among gene pairs within threshold 0.4 for each species. The *x*-axis represents the percentage number of syntelog pairs with the query species and *y*-axis represents the target species compared

An analysis across promoter region of syntelog genes showed similarities and differences in the pattern of TFBS. ADASS algorithm was employed to compare TFBS architecture from different genes in a pairwise manner. A distance score is then assigned for each pair of genes on the basis of matches and mismatches of TFBS patterns in the promoter region. The distance scores vary from 0 (highly similar) to 1 (highly divergent) for a pair of sequences. A comparison of TFBS architecture between *F. vesca* and *M. domestica* syntelogs showed around 30% of the genes were present within 0.5 score, suggesting similar patterns (Additional file 6a). Further, we expanded this analysis across other Rosaceae species. For *F. vesca* gene promoters with other species, a significant number of genes showed similar binding site patterns in the promoters (Additional file 6b). For *M. domestica* with other species showed comparatively higher conservation than *F. vesca* with rest of the species (Additional file 6c). A threshold 0.4 was set to identify similar architecture among syntelogs. The percentage of genes that fall within threshold 0.4 for each species is shown in (Fig. 4). A distance tree was constructed to analyse the clustering of similar architecture. The ADASS score for all species was used to generate the distance score matrix and used to cluster similar sequences for all syntelogs. A distance tree was constructed from the matrix using NJ method in Phylip package and was viewed in Dendroscope (Additional file 7). TFBS architecture compared between each syntelogs, showed many clusters. In most of the clusters, we could see syntelogs clustered together from an evolutionarily closely related species. A majority of them are from same subspecies and very few of them shown with different subspecies from Rosaceae family. We looked at highly similar syntelog genes from *F. vesca* and *M. domestica*. Many genes had an ADASS score of less than 0.1, indicating that they would have conserved cis elements in the promoter region. The table (Additional file 8) shows the top 50 such genes, which can be used as candidate genes for future research.

### AP2/ERF gene family analysis

**Fig. 5:**
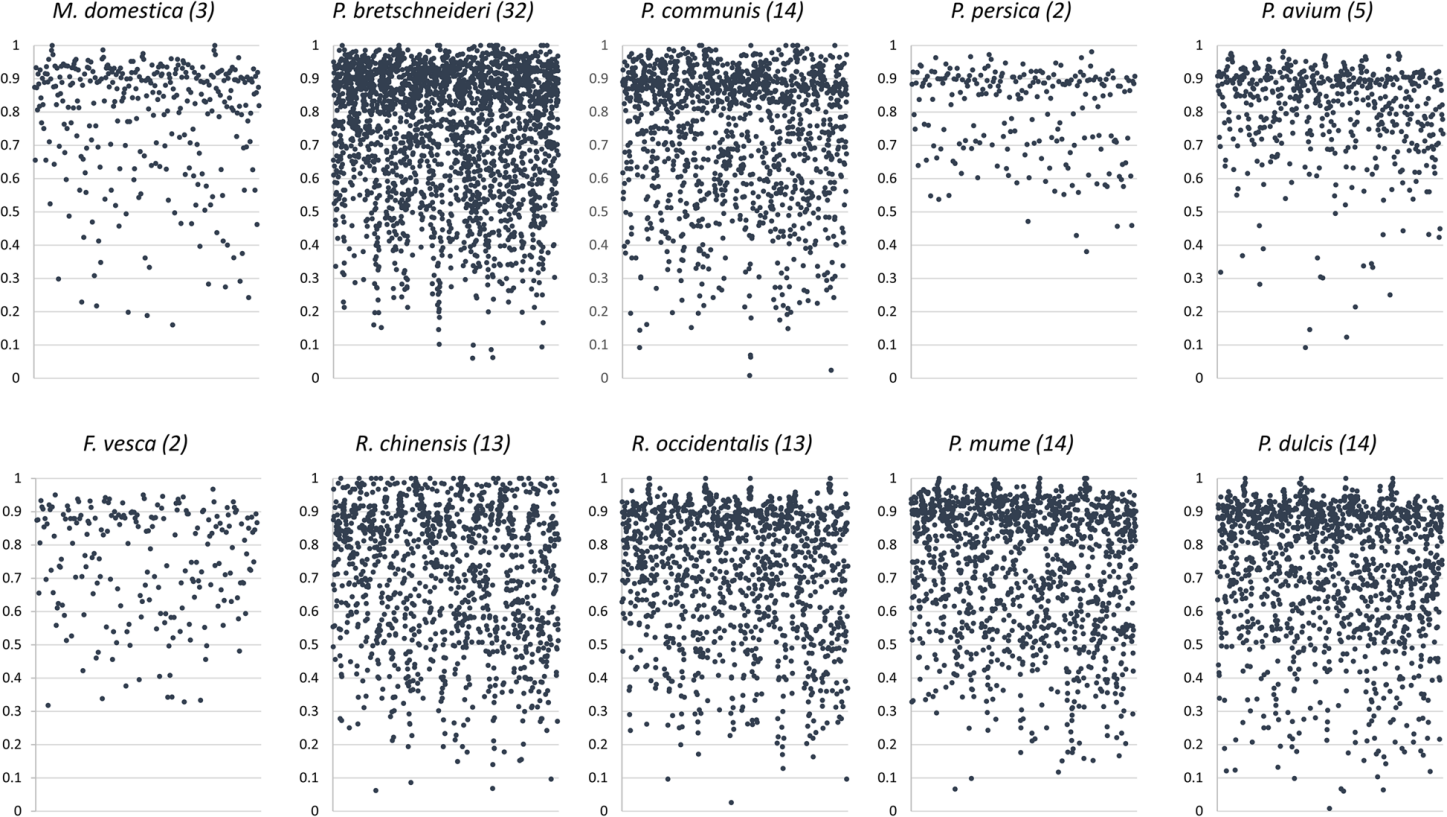
A case study on AP2/ERF family of syntelog genes. Scatter plots showing inter species ADASS scores for gene pairs between Rosaceae species. Number of genes from AP2/ERF syntelog groups for each species have been given in the bracket. The scores vary from zero (identical TFBS patterns) to one (completely different TFBS patterns). The *x*-axis represents the number of syntelog pairs and *y*-axis represents pairwise scores

One of the largest groups of TFs families, AP2/ERF genes are involved in the regulation of biotic and abiotic stress responses. This family is characterised by a conserved AP2-DNA binding domain. The AP2 sub-family encodes for TFs with two AP2 domains and known to regulate developmental process of plants. While the ERF and DREB proteins having a single AP2 domain are the key regulators in response to biotic and abiotic stress. Two groups out of 32 were identified as AP2/ERF family. These groups include 121 syntelog genes from *F. vesca* (2), *M. domestica* (3), *P. avium* (5), *P. bretschneideri* (32)*, P. communis* (14)*, P. dulcis* (14), *P. mume* (14)*, P. persica* (2), *R. chinensis* (13) and *R. occidentalis* (13). Domain analysis showed only one sequence from *R. occidentalis* has two AP2 domains, while all other members have a single AP2 domain (Additional file 9). Both genes from *F. vesca* were located on chromosome 6 and *M. domestica* genes were at 4, 8 and 9 chromosomes. Synteny analysis using these sequences showed the organisation of syntelogs in various chromosomes of other Rosaceae species (Additional file 10). The DREB TFs activate multiple cold-regulated genes (CORs) by interacting with DRE/CRT elements, present in the promoters. We analysed the promoter sequence of these 121 genes. The TFBS architecture from each gene was compared using ADASS algorithm. A scatter plot was generated using the distance score (Fig. 5) for each species. Inter-species analysis showed comparatively less similar TFBS pattern within this gene family. Less number of gene pairs were retained when given a threshold of 0.4. This conveys that, although being syntelogs from the same gene family, the binding site patterns in the promoter are substantially different.

## Discussions

Rosaceae family members typically grow in cold condition and often subjected to cold stress tolerance. It is important to study the cold tolerance mechanism and the genes involved in the stress tolerance for these plants. In this study, we used computational approaches to identify and analyse putative cold stress responsive genes and their transcription factor binding sites in the promoter of Rosaceae plants. We obtained differentially upregulated gene information for cold stress from apple and strawberry to identify putative genes in eight other Rosaceae family species. A functional annotation of these DEGs showed a variety of gene families such as transcription factors, cytochromes, kinases, transferases and membrane proteins. Majorities of the genes were transcription factors and most of them were from the groups of AP2/ERF and MYB transcription factor families. For other species, genes evolved from a common ancestor were traced using synteny analysis. There were a total of 1469 syntelogs from all ten species that were analysed in detail. When we compared the number of syntelogs predicted from Maloideae (2n = 34), Prunoideae (2n = 16) and Rosoideae (2n = 14) subfamily species, we noticed a direct correlation with genome size and chromosome number. Higher number of syntelogs were identified from Maloidae species. A high number of syntelogs were identified in both *P. bretschneideri* and *P. communis* from Maloideae subgroup compared to other species. Evolution of protein-coding gene families happens through events like WGD or segmental duplication, tandem duplication, and chromosomal and gene rearrangements. We observed more number of dispersed duplication events in *M. domestica,* could be due to recent WGD in Maloideae clade (30–45 MYA) compared to other plants [24]. Apart from the WGD events, other duplication events (like tandem, dispersed and segmental) have contributed to the repertoire of this syntelogs in these species. This suggests that evolutionarily cold stress response gene pool would have expanded and contributes to the cold survival among the Rosaceae family plants.

A function annotation and enrichment analysis of these potential genes showed many transcription factors in these groups, which play a significant role in plant development and stress tolerance. They act as regulatory proteins by regulating a set of targeted genes in a coordinated manner and consequently enhance the stress tolerance of the plant. AP2/ERF is an important transcription factor family that has a major role in response to cold stress. So far, many cold stress responsive genes and their gene regulatory network have been reported in plants. ICE-CBF-COR pathway is one of the most studied pathway related to cold stress in plant crops [25]. CBF, a member of the AP2/ ERF family of transcription factors, are expressed in response to cold temperatures, which in turn, activates many downstream genes that leads to cold acclimation chilling and freeze tolerance in plants [26]. Apart from these key regulators, many other TF families such as bHLH, WRKY, NAC and MYB also known to helps in regulating the gene expression under cold stress.

A *cis*-element is required in the promoters of stress-responsive genes for the expression under specific stress. The gene promoter analysis using STIFAL identified and classified popular abiotic stress transcription factor-binding sites for these putative cold stress response genes. There are 19 such models of *cis*-elements in STIFAL, based on abiotic stress response transcription factor families, which were built as HMMs and were validated using Jack-knifing method [27]. STIFAL predicted a total of 11,145 TFBSs from the promoter sequence of 1408 syntelog genes. MYB is the largest and diverse group of TFs and often co-occur with other TFs. Hence, MYB classes were most abundant followed by bHLH, WRKY and AP2/ERF TF families. However, the trend remains almost similar when compared the occurrences of TFBS between Rosaceae species. CBF or DREB transcription factors, which belongs to AP2/ERF family, is the key regulator in the pathway, which binds to the DRE or CRT *cis*-elements in the promoter of CORs. The abundance of this important cold regulated transcription factor family in the dataset was revealed by functional annotation and enrichment analysis. Aside from the AP2/ERF family, other TF families known to be involved in the cold stress response include WRKY, bHLH, bZIP, MYB, and NAC [28]. In our analysis, we observed that these *cis*-elements are highly enriched in the promoter region. MYB was the most abundant TFBS found in almost all gene promoters. Following MYB, the presence of other TFBS in bHLH (991), WRKY (901) AP2/EREBP (776), bZIP (735) and NAC (634) suggests that these TF families are important for cold stress tolerance in these plants. Separately, we noticed a few gene promoters that are enriched with various group of TF families, which could play role in multiple stress response or other functional roles. PP2C-type protein phosphatase gene from *P. dulcis* (Prudul26A011712P1) predicted 34 various TFBSs in 1000 bp promoter sequence. This includes MYB (20), NAC (2), AP2 (2), WRKY (2), ARF (2), bHLH (2) and HSF (4) TF family binding sites. Another gene, serine/threonine-protein kinase from *P. dulcis* (Prudul26A014996P1) predicted 32 *cis*-regulatory elements including MYB (7), NAC (4), AP2 (1), WRKY (4), ARF (6), bHLH (6), HSF (3) and bZIP (1). Apart from highly abundant MYB binding sites, tandemly repeated AP2 binding sites were observed in many of the promoters. It will be interesting to investigate the role of these genes in response to stress.

Further, we noticed few sequences from different Rosaceae species sharing highly similar promoter sequences. The TFBS pattern was conserved among those syntelogs. A higher amount of conservation was observed in closely related species in terms of position and combination of TFBS. Cytochrome p450 genes from Maloideae species *P. communis* and *P. bretschneideri* showed similar TFBS architecture (AP2 ~ AP2 ~ MYB ~ MYB ~ AP2 ~ AP2 ~ bHLH~MYB ~ MYB ~ MYB ~ HSF). Gene duplication events must have played a role in this conservation among closely related Rosaceae family species. There are also instances of similarities between different subfamily species, such as *P. communis* (pycom09g00070), a cytochrome p450 gene with Hypostatin resistance gene from *P. dulcis* (Prudul26A022009P1). These two different species genes showed same promoter TFBS architecture (WRKY~MYB ~ MYB ~ MYB ~ MYB ~ AP2). These similarities and differences in TFBS architecture in each syntelogs were further studied using an in-house algorithm ADASS. Overall, we find that for most of the species, *M. domestica* and *R. occidentalis* have higher percentage of association. Whereas, *P. avium* showed less association with *M. domestica* compared to other species. Even though the number of syntelogs were less in *P. persica*, it showed higher percentage with *M. domestica*. This analysis suggest that the trend is almost similar when we see the percentage of similar gene promoter sequences within threshold 0.4 across Rosaceae species.

There have been recent WGD events in the Maloideae and Prunoideae clades, therefore we can expect at the genome level. We noticed few TFBS patterns within same subfamily species were similar, whereas the patterns among syntelogs were divergent when compared across other subfamilies from Rosaceae species. This indicates that the similarity in the promoter region of the syntelog genes could be proportional to the evolutionary distance of the species. Our study overall suggests a novel method for identifying potential target genes in biotic and abiotic stress research. It also provides information on key genes for a large number of species within or across plant families. This analysis can be used to investigate the crosstalk between TFs and other important genes.

## Conclusions

In this study, we conducted a comparative genome wide study for putative cold stress-response genes in ten Rosaceae species. Our in silico study reveals useful information about expanded pool of cold-responsive genes and abundance of popular transcription factor binding sites in the upstream of such genes in the Rosaceae family species. Synteny analysis from apple and strawberry identified syntelog groups containing putative cold stress response genes from all species. Using WGD analysis, the number of syntelogs associated with the species evolutionary distance. Putative binding sites in the promoters of these genes were identified, and their conservation across species was investigated using computational algorithms. The information of putative cold stress responsive genes from Rosaceae family allows further studies for understanding the mechanism, regulation by TF binding and molecules involved in cold response.

## Methods

### Collection of DEGs and genome data

A literature survey was carried out to obtain differentially expressed genes (DEGs) under cold stress from Rosaceae family species. Cold stress upregulated genes for *Fragaria vesca* (Strawberry) and *Malus domestica* (Apple) was collected from Zhang et al. [21] and Du et al. [22], respectively. The genome information of *M. domestica* [29], *F. vesca* [30], *Prunus avium* (Sweet cherry) [31], *Prunus dulcis* (Almond) [32], *Prunus persica* (Peach) [33], *Pyrus bretschneideri* (Chinese pear) [24], *Pyrus communis* (European pear) [34], *Rosa chinensis* (Chinese rose) [35], and *Rubus occidentalis* (Black raspberry) [36] were obtained from Genome Database for Rosaceae (GDR) [37] (http://www.rosaceae.org/), and the *Prunus mume* (Chinese plum) genome [38] sequence was obtained from NCBI repository (https://www.ncbi.nlm.nih.gov/genome/?term=txid102107[orgn]). A species tree was generated and the divergence time was obtained using online tool TimeTree [39].

### Synteny and duplication analysis

Synteny analysis was performed to investigate collinear blocks between the chromosomes of Rosaceae species. First, all versus all BLASTP [40] with an E-value threshold 1.0E-5 was performed to predict potential homologous gene pairs in Rosaceae species. DEGs obtained for *M. domestica* and *F. vesca* from literature were used as input. Predicted homologs location in the chromosome for corresponding plants were obtained from the genome annotation data. Collinear blocks between Rosaceae species were detected using MCScanX package [41]. Conserved collinear blocks were visualized with the web based VGSC (Vector Graph toolkit of genome Synteny and Collinearity) [42]. Different types of duplication events (Tandem, Proximal, Dispersed and WGD/Segmental) were further estimated using duplicate gene classifier module of MCScanX program.

### Function annotation and enrichment analysis

Function annotation of syntelog genes was carried out using BLASTP program [43] against Viridiplantae database from Uniprot [44]. GO terms [45] were obtained from homologous sequences to understand basic set of biological process and molecular function mediated by these genes. Further, an enrichment analysis was performed using DAVID [46] and scatter plot was generated using REViGO visualization tool [47]. The domain composition of each syntelog gene was studied using a java based tool Domosaic [48]. An E-value threshold 1.0E-5 was given for HMM search against Pfam database [49].

### Promoter *ci*s-elements analysis

The chromosome location and gene co-ordinates for the syntelog genes were obtained from genome annotation data obtained from GDR, JGI and NCBI. Thousand base pair upstream region for the syntelogs was extracted using gene co-ordinates information. Promoter region was extracted for both forward and reverse orientations of the gene in the strands (for reverse direction, reverse complement of the sequence has been used). STIFAL, an algorithm [50] to predict popular abiotic stress responsive transcription factor binding sites in the promoter of plant gene, was used to identify potential binding sites. It uses Hidden Markov Models (HMMs) of nucleotide binding site patterns of *cis*-elements that are well known for stress response in plants. One thousand base pair upstream region of the genes was provided as input to STIFAL server (http://caps.ncbs.res.in/stif/). A Z-score threshold ≥1.5 was applied to filter out false positive TFBS hits [27]. Each predicted hits were further classified into different TF family classes.

### Analysis of TFBS in the promoter region

Alignment-free domain architecture similarity search (ADASS) [51], originally used for the comparison and analysis of domain architectures, was used to analyse the similarities in TFBS pattern among a pair of syntelog promoter sequence. Here, each predicted TFBS in the upstream sequence was provided as discrete units into ADASS, in order to classify proteins according to similarity in the predicted TFBS patterns. For each gene, a TFBS architecture was derived from STIFAL output and used as input for ADASS algorithm. A distance matrix was constructed using ADASS algorithm by comparing all the TFBS architectures. ADASS divides the architectures into all possible triplets and, compares compare between a pair of architecture. For each triplet compared, distance scores were assigned based on events like shuffling, duplication and inversion and the cumulative score is calculated for each pair of TFBS architecture. PHYLIP [52] was used to construct a distance tree using the score matrix from ADASS and viewed using Dendroscope [53].

## Supplementary Information

**Additional file 1: Supplementary Table S1.** Details of plants from Rosaceae family considered in this study.

**Additional file 2: Supplementary Table S2.** Functions predicted for DEGs obtained from literature for *F. vesca* and *M. domestica*.

**Additional file 3: Supplementary Table S3.** Chromosome information of syntelog genes identified from Rosaceae species.

**Additional file 4: Supplementary Table S4.** Syntelog genes distribution in 32 gene family groups. Each groups include one or more genes from different Rosaceae species.

**Additional file 5: Supplementary Table S5.** Popular transcription factor binding sites predicted for each syntelog genes using STIFAL. A z-score threshold of 1.5 was applied to filter out false positive hits. The position of binding sites in the 1000 bp promoter region has been included in the table.

**Additional file 6: Supplementary Fig. S1.** Graphs showing the distribution of ADASS scores among syntelog gene pairs. a) Gene pairs between *F. vesca and M. domestica* with other Rosaceae species syntelogs, b) gene pairs for *F. vesca* with other Rosaceae species syntelogs, c) gene pairs for *M. domestica* with other Rosaceae species syntelogs. The scores vary from zero (identical TFBS patterns) to one (completely different TFBS patterns). The *x*-axis represents the number of syntelog pairs and *y*-axis represents pairwise scores.

**Additional file 7: Supplementary File S1.** Distance tree constructed using ADASS algorithm: Similar sequences clustered together. The genes have been named according to the syntelog group number.

**Additional file 8: Supplementary Table S6.** The top 50 highly similar syntelog genes for *F. vesca* and *M. domestica* in other species were chosen based on their ADASS score.

**Additional file 9: Supplementary File S2.** Pfam domain architecture for AP2/ERF family syntelog genes generated using Domosaic. Two groups of AP2/ERF family genes have been shown with a central AP2 domain.

**Additional file 10: Supplementary Fig. S2.** Circos plot showing distribution of AP2/ERF syntelog group genes in the chromosomes of different species. Two groups of AP2/ERF genes have been plotted separately.

## Data Availability

Genome data are available at public repositories such as Genome database of Rosaceae (*Fragaria vesca*: https://www.rosaceae.org/species/fragaria/fragaria_vesca/genome_v1.1; *Rosa chinensis*: https://www.rosaceae.org/species/rosa/chinensis/genome_v1.0; *Rubus occidentalis*: https://www.rosaceae.org/analysis/268; *Malus domestica*: https://www.rosaceae.org/species/malus/malus_x_domestica/genome_GDDH13_v1.1; *Pyrus bretschneideri*: https://www.rosaceae.org/species/pyrus_bretschneideri/genome_v1.1; *Pyrus communis*: https://www.rosaceae.org/species/pyrus/pyrus_communis/genome_v2.0; *Prunus avium*: https://www.rosaceae.org/species/prunus_avium/genome_v1.0.a1; *Prunus dulcis*: https://www.rosaceae.org/species/prunus/prunus_dulsis/lauranne/genome_v1.0); (*Prunus persica*: https://www.rosaceae.org/species/prunus_persica/genome_v2.0.a1) and NCBI (*Prunus mume*: https://www.ncbi.nlm.nih.gov/genome/?term=txid102107[orgn]).

## Abbreviations

TFBS: Transcription factor binding site
DRE/CRT/LTRE: Dehydration responsive element/C-repeat/Low temperature responsive element)
CBF/DREBP: C-repeat binding factor/dehydration-responsive element binding protein
CORs: Cold responsive genes
WGD: Whole genome duplication
HMM: Hidden markov model
MYB: Myeloblastosis
AP2/ERF: APTELA2/Ethylene responsive factor
EREBP: Ethylene responsive element binding protein
bHLH: Basic helix loop helix
bZIP: Basic leucine zipper
NAC (NAM): No apical meristem
ARF: Auxin response factor
HB: Homeobox
HSF: Heat shock factor
ABI3: Abscisic acid insensitive3
ABRE: Abscisic acid response element
Znf: Zinc finger
SBP: Squamosa promoter binding protein
