# Supplementary figures and images for: Computational analysis of potential candidate genes involved in the cold stress response of ten Rosaceae members
Source: BMC Genomics. 2022 Jul 16;23:516. doi: 10.1186/s12864-022-08751-x (PMC9288012; doi:10.1186/s12864-022-08751-x)

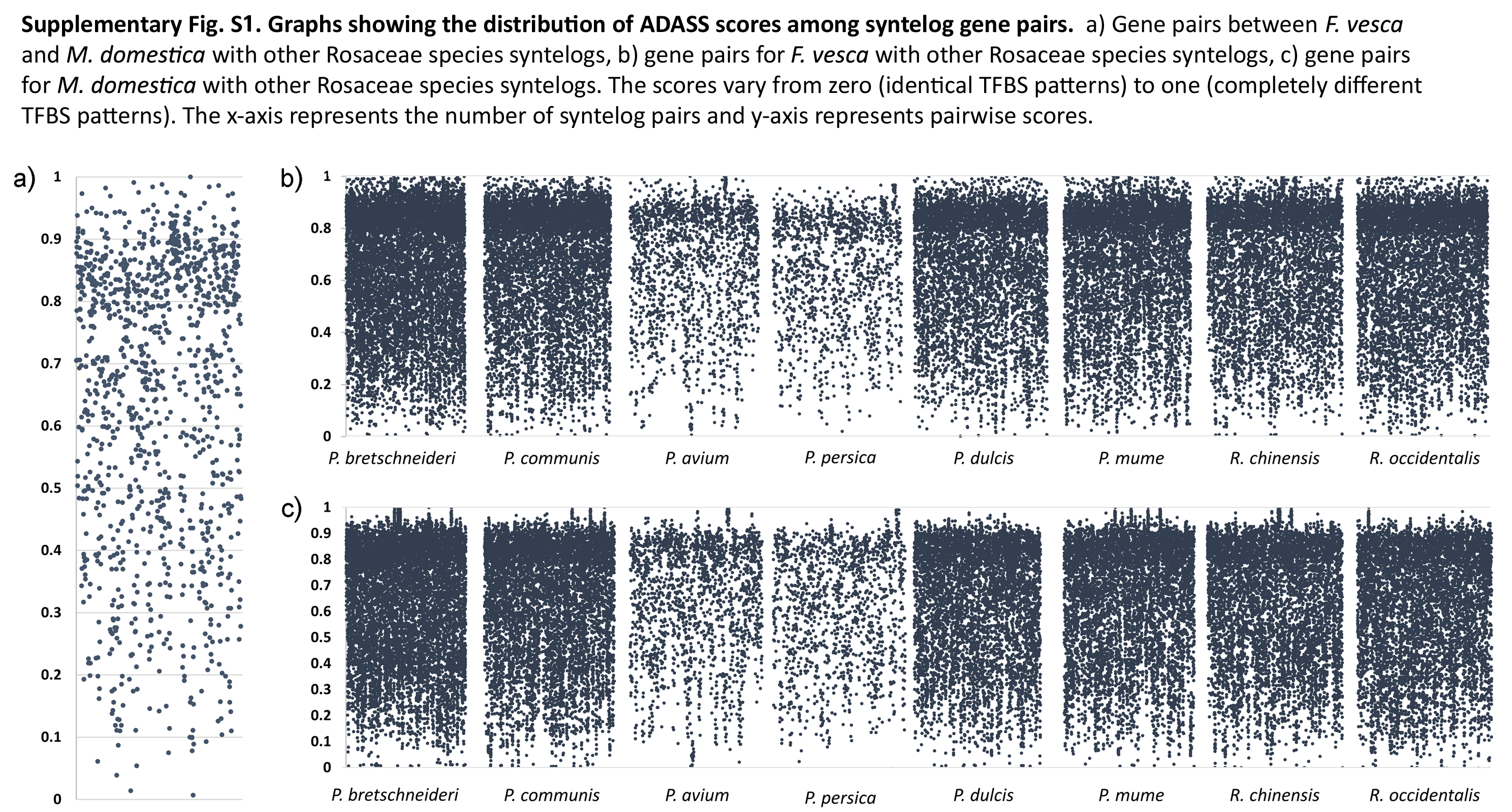

Supplement: Supplementary file 6 — Additional file 6: Supplementary Fig. S1. Graphs showing the distribution of ADASS scores among syntelog gene pairs. a) Gene pairs between F. vesca and M. domestica with other Rosaceae species syntelogs, b) gene pairs for F. vesca with other Rosaceae species syntelogs, c) gene pairs for M. domestica with other Rosaceae species syntelogs. The scores vary from zero (identical TFBS patterns) to one (completely different TFBS patterns). The x-axis represents the number of syntelog pairs and y-axis represents pairwise scores. [file 12864_2022_8751_MOESM6_ESM.tif]

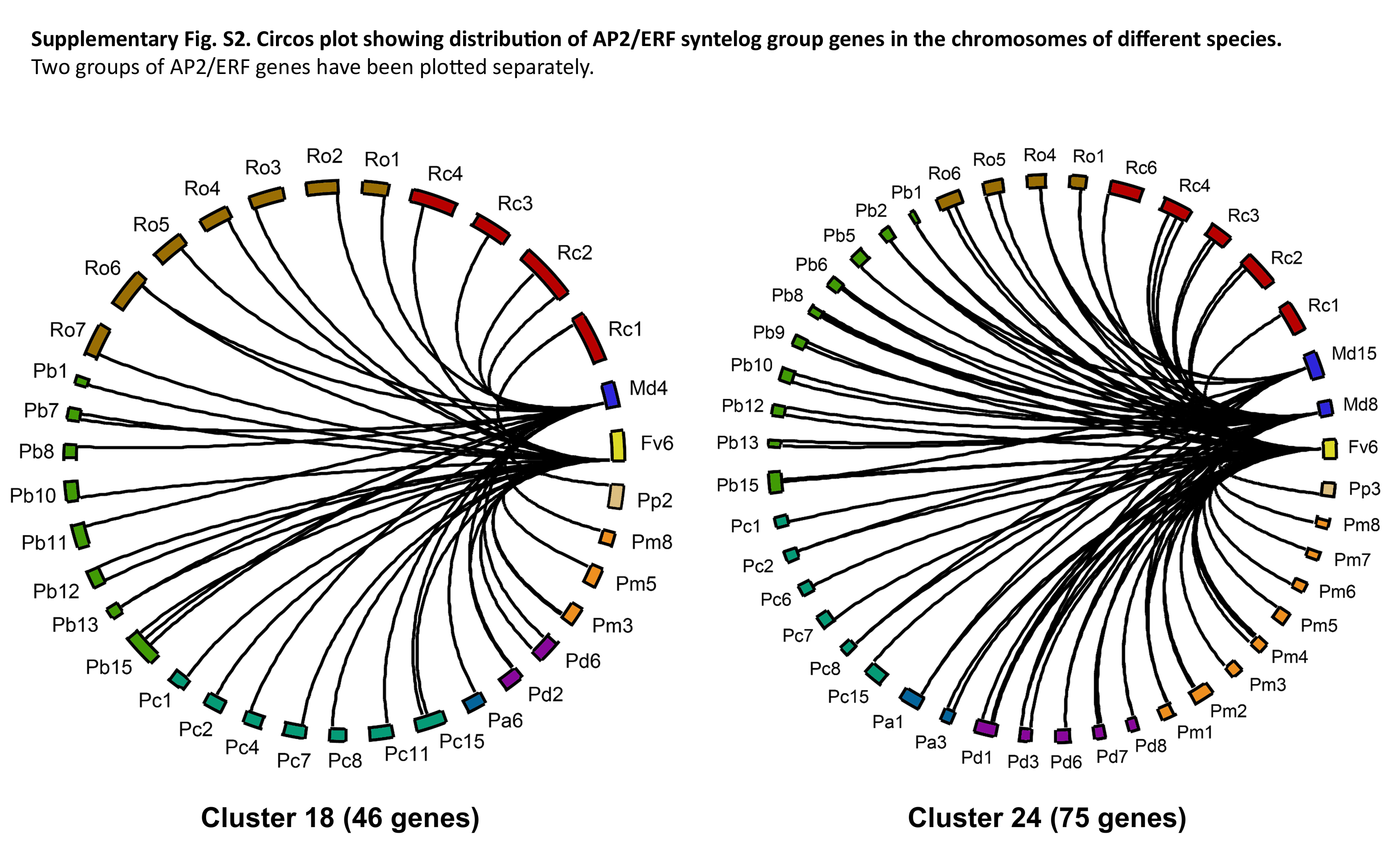

Supplement: Supplementary file 10 — Additional file 10: Supplementary Fig. S2. Circos plot showing distribution of AP2/ERF syntelog group genes in the chromosomes of different species. Two groups of AP2/ERF genes have been plotted separately. [file 12864_2022_8751_MOESM10_ESM.tif]
