# Supplementary material for: Computational analysis of potential candidate genes involved in the cold stress response of ten Rosaceae members
Source: BMC Genomics. 2022 Jul 16;23:516. doi: 10.1186/s12864-022-08751-x (PMC9288012; doi:10.1186/s12864-022-08751-x)
Supplement: Supplementary file 9 — Additional file 9: Supplementary File S2. Pfam domain architecture for AP2/ERF family syntelog genes generated using Domosaic. Two groups of AP2/ERF family genes have been shown with a central AP2 domain. [file 12864_2022_8751_MOESM9_ESM.pdf]

Supplementary File S2. Pfam domain architecture for AP2/ERF family syntelog genes generated using Domosaic. Two groups of AP2/ERF family genes have been shown with a central AP2 domain.

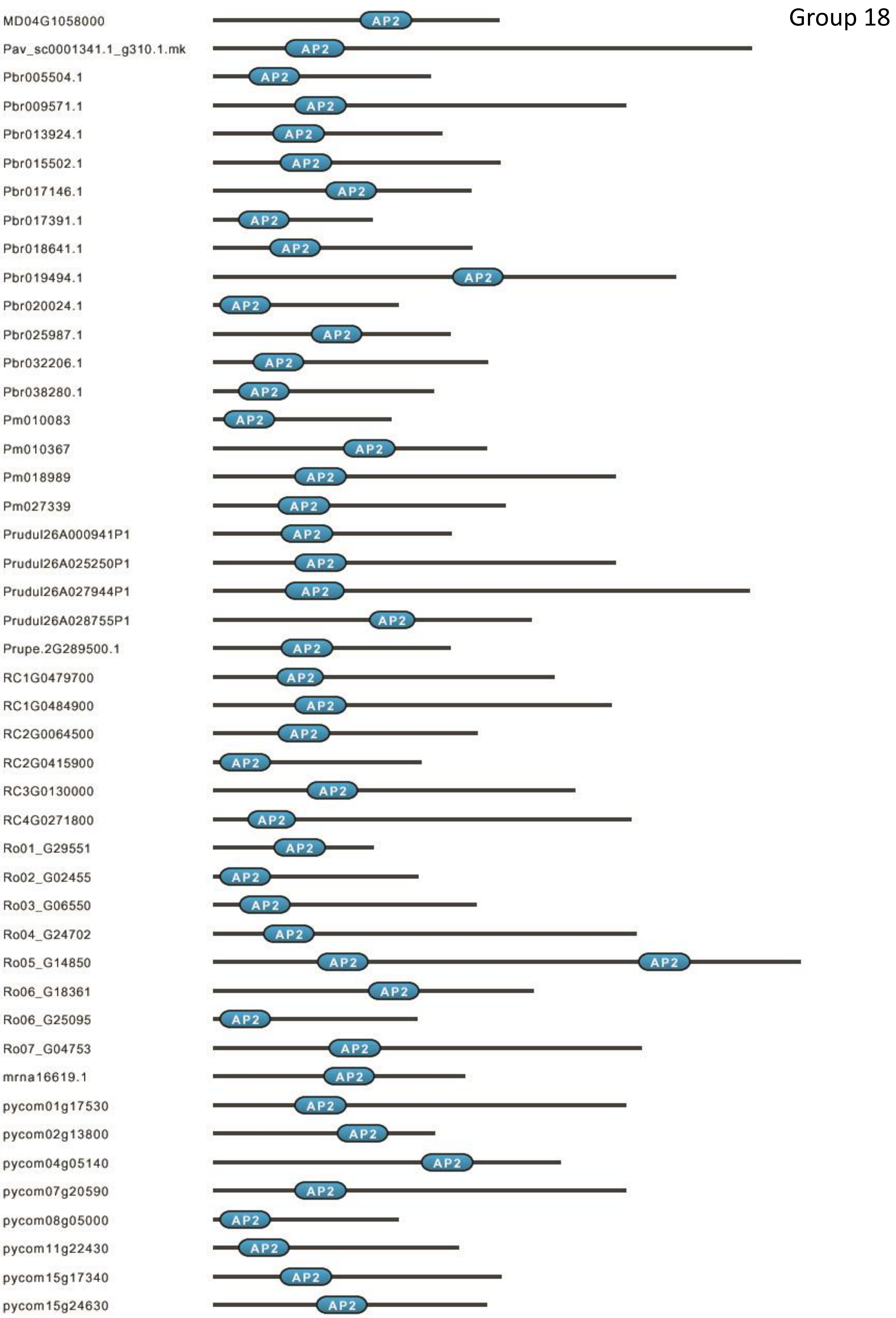

Group 24

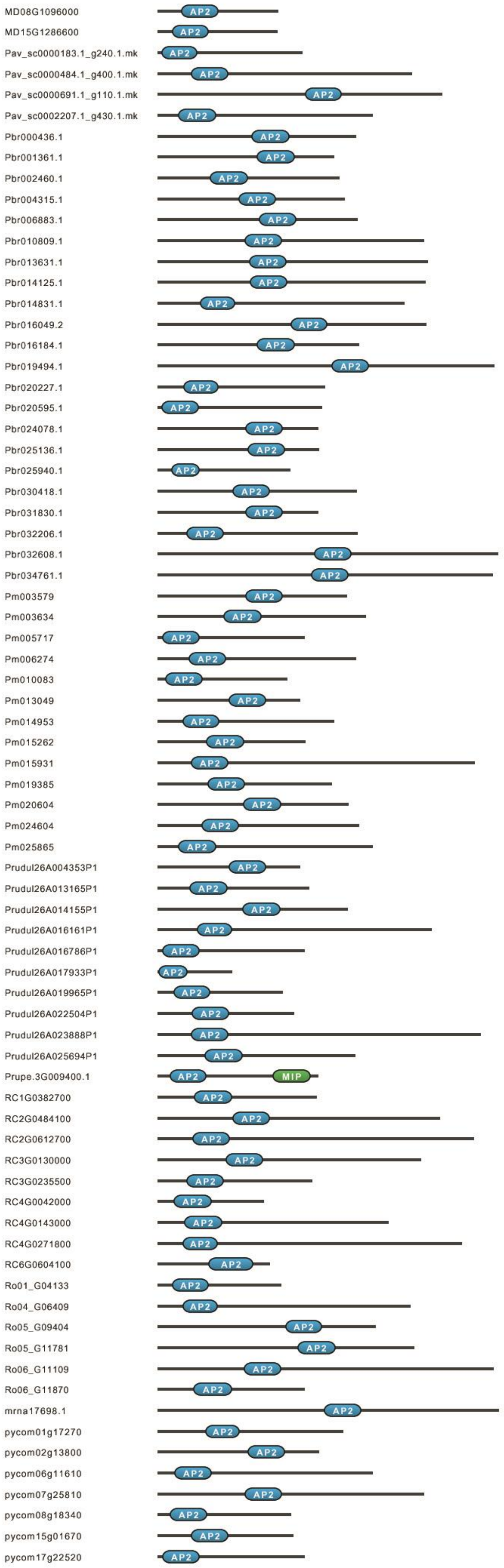
MD08G1096000 AP2
MD15G1286600 AP2
Pav_sc0000183.1_g240.1.mk AP2
Pav_sc0000484.1_g400.1.mk AP2
Pav_sc0000691.1_g110.1.mk AP2
Pav_sc0002207.1_g430.1.mk AP2
Pbr000436.1 AP2
Pbr001361.1 AP2
Pbr002460.1 AP2
Pbr004315.1 AP2
Pbr006883.1 AP2
Pbr010809.1 AP2
Pbr013631.1 AP2
Pbr014125.1 AP2
Pbr014831.1 AP2
Pbr016049.2 AP2
Pbr016184.1 AP2
Pbr019494.1 AP2
Pbr020227.1 AP2
Pbr020595.1 AP2
Pbr024078.1 AP2
Pbr025136.1 AP2
Pbr025940.1 AP2
Pbr030418.1 AP2
Pbr031830.1 AP2
Pbr032206.1 AP2
Pbr032608.1 AP2
Pbr034761.1 AP2
Pm003579 AP2
Pm003634 AP2
Pm005717 AP2
Pm006274 AP2
Pm010083 AP2
Pm013049 AP2
Pm014953 AP2
Pm015262 AP2
Pm015931 AP2
Pm019385 AP2
Pm020604 AP2
Pm024604 AP2
Pm025865 AP2
Prudul26A004353P1 AP2
Prudul26A013165P1 AP2
Prudul26A014155P1 AP2
Prudul26A016161P1 AP2
Prudul26A016786P1 AP2
Prudul26A017933P1 AP2
Prudul26A019965P1 AP2
Prudul26A022504P1 AP2
Prudul26A023888P1 AP2
Prudul26A025694P1 AP2
Prupe.3G009400.1 AP2 MIP
RC1G0382700 AP2
RC2G0484100 AP2
RC2G0612700 AP2
RC3G0130000 AP2
RC3G0235500 AP2
RC4G0042000 AP2
RC4G0143000 AP2
RC4G0271800 AP2
RC6G0604100 AP2
Ro01_G04133 AP2
Ro04_G06409 AP2
Ro05_G09404 AP2
Ro05_G11781 AP2
Ro06_G11109 AP2
Ro06_G11870 AP2
mrna17698.1 AP2
pycom01g17270 AP2
pycom02g13800 AP2
pycom06g11610 AP2
pycom07g25810 AP2
pycom08g18340 AP2
pycom15g01670 AP2
pycom17g22520 AP2
