# Supplementary material for: Computational analysis of potential candidate genes involved in the cold stress response of ten Rosaceae members
Source: BMC Genomics. 2022 Jul 16;23:516. doi: 10.1186/s12864-022-08751-x (PMC9288012; doi:10.1186/s12864-022-08751-x)

Supplementary File S1. Distance tree constructed using ADASS algorithm: Similar sequences clustered together. The genes have been named according to the syntelog group number.

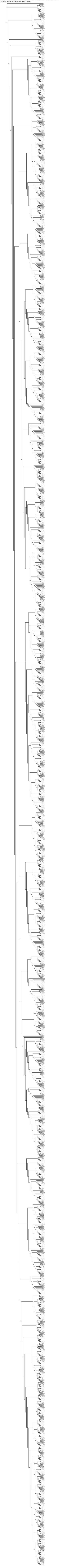

Supplement: Supplementary file 7 — Additional file 7: Supplementary File S1. Distance tree constructed using ADASS algorithm: Similar sequences clustered together. The genes have been named according to the syntelog group number. [file 12864_2022_8751_MOESM7_ESM.pdf]
